# Development of a Phage Cocktail to Target *Salmonella* Strains Associated with Swine

**DOI:** 10.3390/ph15010058

**Published:** 2022-01-02

**Authors:** Anisha M. Thanki, Viviana Clavijo, Kit Healy, Rachael C. Wilkinson, Thomas Sicheritz-Pontén, Andrew D. Millard, Martha R. J. Clokie

**Affiliations:** 1 Department of Genetics and Genome Biology, College of Life Sciences, University of Leicester, Leicester LE1 7RH, UK; at2632@leicester.ac.uk (A.M.T.); kithealy94@gmail.com (K.H.); adm39@leicester.ac.uk (A.D.M.); 2 Department of Biological Sciences, Universidad de los Andes, Bogota 111711, Colombia; in-clavi@uniandes.edu.co; 3 Swansea University Medical School, Swansea University, Swansea SA2 8PP, UK; r.c.wilkinson@swansea.ac.uk; 4 Section for Evolutionary Genomics, The GLOBE Institute, University of Copenhagen, 1353 Copenhagen, Denmark; thomassp@sund.ku.dk; 5 Centre of Excellence for Omics-Driven Computational Biodiscovery, AIMST University, Bedong 08100, Malaysia

**Keywords:** *Salmonella* phages, phage cocktails, phage therapy, phage characterisation, larvae infection model, single nucleotide polymorphisms

## Abstract

Infections caused by multidrug resistant *Salmonella* strains are problematic in swine and are entering human food chains. Bacteriophages (phages) could be used to complement or replace antibiotics to reduce infection within swine. Here, we extensively characterised six broad host range lytic *Salmonella* phages, with the aim of developing a phage cocktail to prevent or treat infection. Intriguingly, the phages tested differed by one to five single nucleotide polymorphisms. However, there were clear phenotypic differences between them, especially in their heat and pH sensitivity. *In vitro* killing assays were conducted to determine the efficacy of phages alone and when combined, and three cocktails reduced bacterial numbers by ~2 × 10^3^ CFU/mL within two hours. These cocktails were tested in larvae challenge studies, and prophylactic treatment with phage cocktail SPFM10-SPFM14 was the most efficient. Phage treatment improved larvae survival to 90% after 72 h versus 3% in the infected untreated group. In 65% of the phage-treated larvae, *Salmonella* counts were below the detection limit, whereas it was isolated from 100% of the infected, untreated larvae group. This study demonstrates that phages effectively reduce *Salmonella* colonisation in larvae, which supports their ability to similarly protect swine.

## 1. Introduction

Non-typhoidal *Salmonella* spp. are a major cause of foodborne gastroenteritis worldwide, causing 155,000 diarrheal related deaths annually [1,2]. In the United Kingdom alone, the bacterium is responsible for 33,000 human infections each year [3], and, on average, 11.7% of these *Salmonella* infections are linked to the ingestion of contaminated pork products [4]. However, using antibiotics to treat and reduce *Salmonella* infections in pigs is becoming increasingly difficult, as disease-causing strains are often resistant to the most common antimicrobials used in veterinary practice [5,6,7]. Consequently, multidrug resistant (MDR) *Salmonella* strains have entered the human food chain and are posing a major health threat [8]. Novel antimicrobials are needed to help treat and control the spread of *Salmonella* from pigs to humans [9].

One promising alternative is the use of bacteriophages (phages), viruses that target and kill bacteria [10,11]. Several studies have shown that phage therapy in pigs is effective and can significantly reduce *Salmonella* colonisation both pre- [12,13,14,15,16,17,18] and post-slaughter [19,20,21]. However, although these studies show great potential for phages to be used in swine, the phages used in the experiments were not fully characterised. In all cases, either genetic data needed to establish that phages are lytic, or phenotypic data such as host range to show if phages lyse representative strains from *Salmonella* serotypes associated with pigs, or both data sets were not presented within the context of efficacy trial data. Furthermore, existing studies did not investigate phage resilience to environmental factors, such as pH and temperature, which is needed to optimise phage formulations for delivery. For example, if phages are added to animal feed they need to survive temperatures above 55 °C for at least twenty minutes potentially up to an hour, dependent on the mill used during the feed pelleting process [22]. In addition, phages could be delivered to pigs via their addition to drinking water and for this it would need to be determined how stable phages are at ambient temperatures and in water. The pH stability is also important to consider as gastrointestinal pH changes could affect phage activity, which in pigs can range between pH 1.8 and pH 8 [23,24]. Finally, further data are needed on whether the phages kill bacteria equally efficiently when used individually or as phage cocktails. Such detailed phage characterisation is needed to improve our understanding of *Salmonella* phages and has been conducted for other phages, such as for phages infecting *Ralstonia solanacearum* [25] and for a collection of phage isolated from sewage [26].

As *Galleria mellonella* larvae have a similar innate immune response as larger vertebrates [27,28], they have been successfully used to test efficacy of phage lysis in vivo [29,30,31,32]. The infection model is both simpler and cheaper and requires a less complex ethical framework than mammalian studies [33], allowing a larger number of formulations, doses and treatment regimens to be tested [34].

This study addresses the generation of phenotypic characterisation data needed for *Salmonella* phage development. We investigate phenotypic behaviour and efficacy against *Salmonella* for phages used individually and when combined in phage cocktails. We investigated six lytic phages SPFM2, SPFM4, SPFM10, SPFM14, SPFM17 and SPFM19 from our *Salmonella* phage collection, which are 99% similar to each other based on average nucleotide identity (ANI0). All six phages are lytic based on the absence of any known lysogeny associated genes identified in the phage genomes, they are members of *Myoviridae* family within *Caudovirales*, and based on the genome data, they are part of the *Seoulvirus* genus [35]. The phages were selected for the study as they have broad host ranges and lyse 98–100% of MDR screened *Salmonella* strains that are representative of the top five serotypes associated with pigs (*n* = 67) [35]. The phages were characterised according to their burst sizes, pH and temperature sensitivity and their bacterial host receptors. Phenotypic differences between the phages were identified, and, subsequently, single-nucleotide polymorphism (SNP) analysis between the phages was conducted to determine if genetic differences can be linked to their phenotypes. To identify optimal phage combinations, killing assay experiments were conducted in vitro and the efficacy of the three best combinations were tested as phage cocktails in challenge larvae studies. This paper showcases data on stability and efficacy of *Salmonella* phage cocktails.

## 2. Results

### 2.1. Phenotypic Parameters of Lifecycle

One step growth curves were carried out on phages: SPFM2, SPFM4, SPFM10, SPFM14, SPFM17 and SPFM19, all of which have a mean burst size of 164 ± 30 PFU/cell. SPFM10 and SPFM17 have a latent period of 20 min and the other phages have latent periods of ~30 min (Supplementary Table S1).

### 2.2. Temperature and pH Stability of Phages

**Figure 1:**
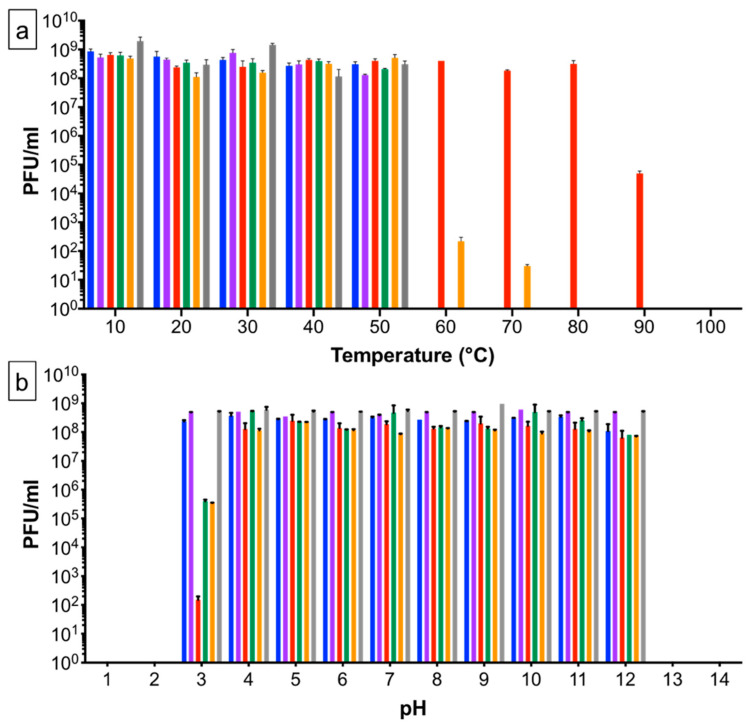
Stability of *Salmonella* phages after being incubated for one hour at a range of different temperatures (**a**) and pH (**b**). Stability of phage SPFM2 is shown in blue, SPFM4 in purple, SPFM10 in red, SPFM14 in green, SPFM17 in orange and SPFM19 in grey. Data presented are the average from three biological replicates, each with three technical repeats. Error bars represent the standard error of the mean (SEM).

To determine virion heat sensitivity, phages were exposed for one hour to temperatures ranging from 4 to 100 °C (Figure 1a). One hour was selected on the basis that when phages are added to feed they would be exposed to high temperatures during the milling process for up to an hour [22,36]. All phages were stable up to 50 °C; SPFM10 and SPFM17 survived at 60 °C and SPFM10 showed no significant drop in titre even at 80 °C. At 90 °C the titre of SPFM10 reduced from 8 × 10^8^ PFU/mL to 6 × 10^4^ PFU/mL. No viable phage particles were recovered at 100 °C. To verify the heat stability of SPFM10 at 90 °C, the phage was incubated in two different temperature-controlled devices: a heat block and a water bath. In both cases, the results were not significantly different (*p* > 0.05), thus confirming the heat stability.

The phage set was exposed to a range of different pH values to determine their sensitivity (Figure 1b), to establish how sensitive the phages would be to pig pH gastrointestinal changes. No phages survived at or below pH 2 or above pH 13. SPFM2, SPFM4 and SPFM19 were stable between pH 3 to 12. SPFM10, SPFM14 and SPFM17 were stable between pH 4–12 and average phage titres remained at the initial 8 × 10^8^ PFU/mL. However, incubation at pH 3 reduced the titre of SPFM10 to 1.5 × 10^2^ PFU/mL, and SPFM14 and SPFM17 to 3.5 × 10^5^ PFU/mL.

### 2.3. Identification of the Bacterial Receptor

To determine the bacterial receptors for the six phages, their efficiency of plating (EOP) was tested on different mutant strains of *Salmonella* strain SL1344 (Table 1. The EOP is a comparison of the phage titre on different strains versus the phages titre on the propagation strain, which is the wild-type SL1344 strain. All phages infected strains with deletion in the flagellar production gene (*flgK*) and in the gene *butB* which encodes the vitamin B12 uptake outer membrane protein [37], as effectively as the wild-type strain SL1344. In contrast, the strain, which had a mutation in *rfaL,* the gene involved in the LPS-related O-antigen production, did not support adsorption or infection and EOP was 0% for all phages (Table 1). This suggests the phages are using a component on LPS as their bacterial receptor.

### 2.4. Genetic Differences between the Six SPFM Phages

Previous analysis suggested that the phages are 99% genetically similar to each other based on ANI [35], but, intriguingly, our results show there are clear phenotypic differences between them. The high sequence conservation between the phages allowed us to identify SNPs that are likely to be linked to these phenotypic differences.

SNP analysis was conducted by comparing the genome of SPFM10 with the clear heat stable phenotype to SPFM2, 4, 14, 17 and 19. There were five SNPs between SPFM10 and the other phages (Table 2 and Supplementary Table S2). One SNP in the genome of SPFM10, which is distinct from the other phages is at position 70,890 bp (locus tag SPFM10_00081), which causes the amino acid change from threonine to serine and results in a non-synonymous change. Further evidence that this SNP confers heat stability can be seen from the observation that this is the only SNP between phages SPFM10 and SPFM14 (a phage with standard heat sensitivity). The largest number of SNPs between SPFM10 and the other phages in the set are seen when compared to SPFM17, five SNPs were identified of which four caused a non-synonymous change and one caused a synonymous change (locus tag SPFM10_00140 at position 113292 bp) (Table 2).

### 2.5. Infection Dynamics of Six SPFM Phages In Vitro

In vitro killing assays were conducted on all phages to determine their individual efficacy to lyse the MDR *S.* Typhimurium S01160-12 strain [38] and when combined as two, three and four-phage cocktails. Phages within the cocktails tested were randomly selected and thirty-six different combinations were tested. All killing assays were assessed over a six hour time period, at a multiplicity of infection (MOI) of 100 to maximise lysis of the target strain, which would help to identify the best phage and phage cocktails. Phage infected CFU counts were compared to an uninfected bacterial control. Below, we discuss CFU counts at one, two and six hours and the results of all phages and phage combinations are discussed in comparison to the uninfected control. All data termed significant are *p* < 0.05.

**Figure 2:**
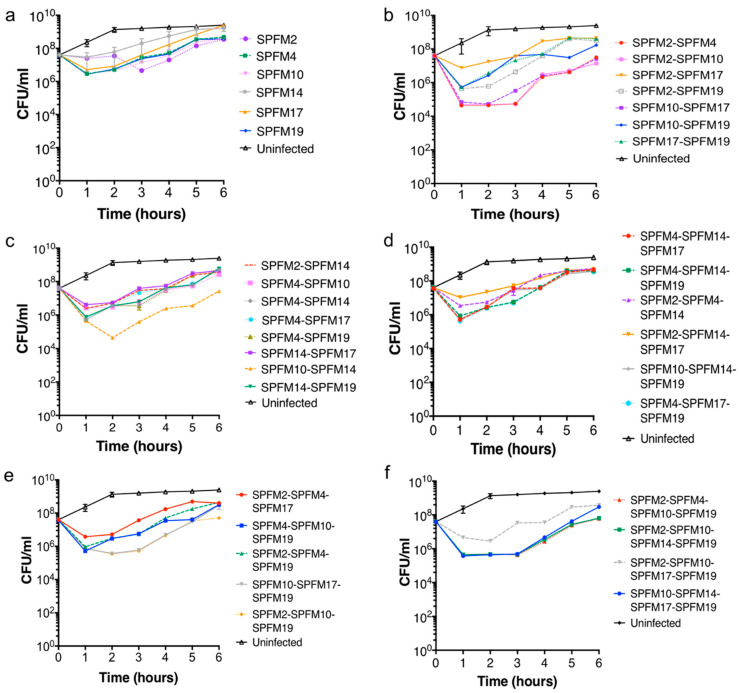
Efficacy of individual (**a**), two (**b**,**c**), three (**d**,**e**) and four (**f**) phage cocktails at reducing an MDR S01160-12 *S*. Typhimurium strain in vitro. Bacterial cultures were infected with the different phage cocktails (**a**–**f**) at MOI of 100 and bacterial counts (CFU/mL) were determined every hour for 6 h. Data presented are averages from three biological repeats, each with three technical repeats and error bars represent SEM.

All phage cocktail combinations tested are listed in Table 3, and killing curves are presented in Figure 2. For clarity, the killing assay results of the two and three phage cocktails are separated in two graphs (Figure 2b–e). In Supplementary Table S3, the average CFU counts are shown. Within the phage cocktails tested, there was a consistent pattern observed with the total phage counts, where after two hours, the phage counts had increased tenfold in comparison to the starting inoculum, indicating phage replication had occurred.

#### 2.5.1. Bacterial killing Assays with Single Phage Suspensions

One hour post-infection, all single phages significantly reduced bacterial counts by 6.2 × 10^1^ to 2 × 10^2^ CFU/mL (Figure 2a). After two hours the average bacterial counts were 1.3 × 10^9^ CFU/mL in the uninfected bacterial culture. In comparison, addition of all single phages significantly reduced bacterial counts. SPFM2, SPFM4, SPFM10 and SPFM19 reduced bacterial counts the most, to an average of 5.5 × 10^6^ CFU/mL. After six hours bacterial counts for cultures infected with SPFM14 and SPFM17 were similar to the bacterial control but cultures infected with the other four phages remained significantly lower.

#### 2.5.2. Efficacy of Multiple Phage Cocktails

All fifteen two-phage cocktail combinations reduced *S*. Typhimurium numbers between 1 × 10^1^ and 1 × 10^3^ CFU/mL one hour post-infection in comparison to the bacterial control (Figure 2b,c). After two hours of incubation, all phage cocktails maintained significant reductions in bacterial counts. Phage combinations SPFM2-SPFM4, SPFM2-SPFM10, SPFM10-SPFM14, and SPFM10-SPFM17 all caused equally high reductions in bacterial numbers two hours post-infection, where average bacteria counts were 4.5 × 10^4^ CFU/mL versus 1.3 × 10^9^ CFU/mL for the bacterial control. At six hours, bacterial regrowth was observed but was significantly lower than the control. Again, the same phage cocktails were most efficient at reducing bacterial counts at six hours. The average bacterial counts were 2.60 × 10^7^ CFU/mL versus 2.50 × 10^9^ CFU/mL for the control.

Eleven three-phage combinations were tested (Figure 2d,e) and all rapidly reduced bacterial numbers by ~3.2 × 10^2^ CFU/mL one hour post-infection (Figure 2d,e). At two hours post-infection, phage cocktails SPFM10-SPFM17-SPFM19 and SPFM2-SPFM10-SPFM19 equally caused the highest reductions of 3.6 × 10^5^ CFU/mL versus 1.3 × 10^9^ CFU/mL for the bacterial control. At six hours post-infection, all phage cocktails maintained significant reductions.

Four four-phage cocktails were tested, and all caused rapid bacterial lysis with a decrease of 3.2 × 10^1^ to 3.2 × 10^2^ CFU/mL one hour post-infection (Figure 2f). After two hours post-infection three cocktails, namely, SPFM10-SPFM14-SPFM17-SPFM19, SPFM2-SPFM10-SPFM14-SPFM19 and SPFM2-SPFM4-SPFM10-SPFM19, were equally the most effective and reduced bacterial counts to 4 × 10^5^ CFU/mL compared to ~1.3 × 10^9^ CFU/mL for the bacterial control. After six hours post-infection, all cocktails maintained significant reductions.

### 2.6. Infection Dynamics of Phage Cocktails In Vivo

The efficacy of the most effective in vitro phage cocktails were tested in the in vivo *G. mellonella* larvae infection model; to determine if they had similar efficacy in a more complex setting. The phage cocktails tested were SPFM10-SPFM14 (two-phage); SPFM4-SPFM10-SPFM19 (three-phage) and SPFM2-SPFM10-SPFM14-SPFM19 (four-phage).

Two phage regimens were tested, to determine which most effectively reduced *Salmonella* colonisation. The first was prophylactic phage administration one hour before larvae were infected with the MDR *S.* Typhimurium S01160-12 strain. The second was concurrent administration of phage when larvae were infected with *Salmonella* (Table 4). All challenge studies were conducted for 72 h, as after which the survival rate of challenged larvae (C-6) dropped to 3%.

#### 2.6.1. Prophylactic Treatment of Phage Cocktails

**Figure 3:**
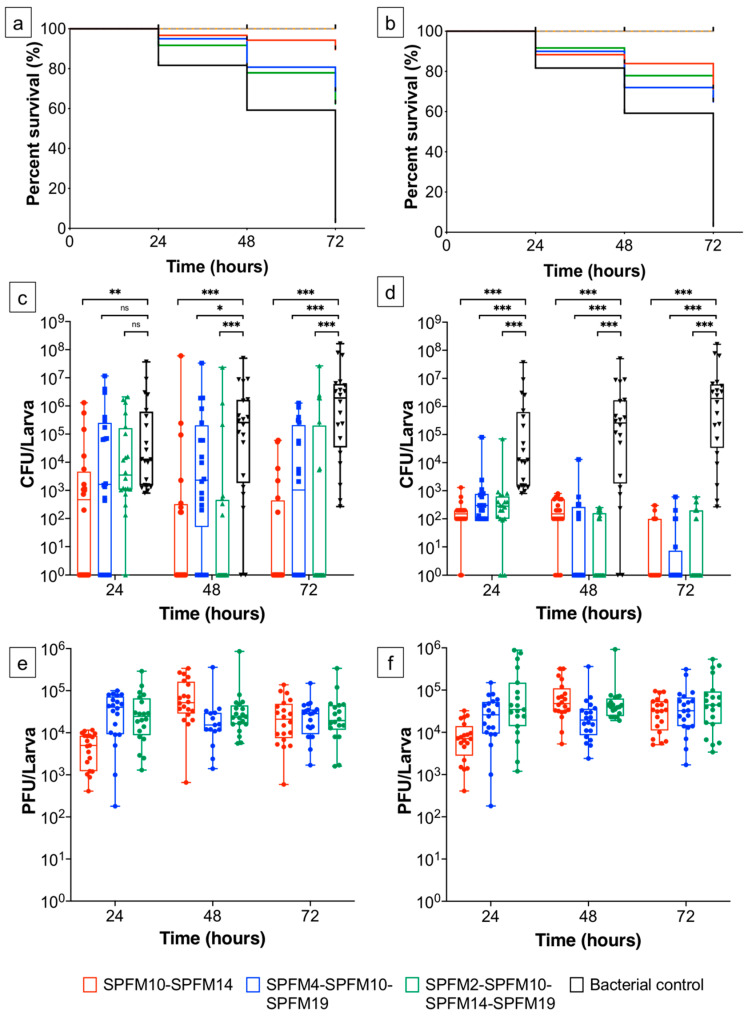
Efficacy of phage cocktails in challenged larvae studies. Larvae were infected with the MDR S01160-12 *S*. Typhimurium strain at 10^5^ CFU/larvae and were either prophylactically treated with phage cocktails one hour prior to infection (**a**,**c**,**e**) or simultaneously administered phage and *Salmonella* (**b**,**d**,**f**). Figures (**a**,**b**) show survival of larvae over 72 h, boxplots (**c**,**d**) show changes in *Salmonella* counts and boxplots (**e**,**f**) show total phage counts of the phage cocktail. The larvae treatment groups were: healthy larvae (orange lines), larvae administered PBS (grey lines), larvae only infected with *Salmonella* (black lines/bars); larvae administered the cocktail SPFM10-SPFM14 (red lines/bars); larvae administered the cocktail SPFM4-SPFM10-SPFM19 (blue lines/bars); and larvae administered the cocktail SPFM2-SPFM10-SPFM14-SPFM19 (green lines/bars). Error bars in graphs (**c**,**d**) show SEM and statistically significant differences between larvae infected with *Salmonella* only (black bars) and treated with phages is displayed on the graphs (*ns* > 0.05, * *p* ≤ 0.05, ** *p* ≤ 0.01 and *** *p* ≤ 0.001).

Larvae prophylactically treated with phage cocktails (Figure 3a,c; Table 4) had mean survival rates of 91% compared to 81% in the control group C-6 (larvae only infected with *Salmonella*) after 24 h. The average bacterial abundance varied between the treatment groups and was 1.0 × 10^2^ (*p* < 0.001), 1.9 × 10^3^ (*p* > 0.05) and 6.3 × 10^3^ CFU/larva (*p* > 0.05) for infected larvae treated with the two (P-2), three (P-3) and four (P-4) phage cocktails, respectively (Table 4). In comparison, group C-6 had higher average bacterial abundance of 4.0 × 10^4^ CFU/larva.

After 48 h, the larvae in group C-6 had average bacterial loads of 6 × 10^5^ CFU/larva and their survival rate dropped to 59% (Figure 3a). In groups P-2, P-3 and P-4 the larvae survival rates were 95%, 81% and 78%, respectively. Additionally, in all phage treatment groups there were significant reductions in *Salmonella* numbers (*p* < 0.05) compared to C-6. Furthermore, *Salmonella* was not recovered (below the limits of detection which is 100 CFU/larva) for 55%, 25% and 65% of larvae in groups P-2, P-3 and P-4, respectively versus isolation of *Salmonella* from 100% of larvae in group C-6.

After 72 h, in group C-6 the bacterial abundance was 3.5 × 10^6^ CFU/larva and survival rates dropped to 3%. In comparison the survival rates in P-2, P-3 and P-4 were 90%, 68% and 62%, respectively and *Salmonella* counts were significantly lower than C-6 (*p* < 0.001). *Salmonella* was not recovered for 70%, 50% and 65% of larvae in groups P-2, P-3 and P-4, respectively, which suggests the phage treatments cleared infection in these larvae. In comparison, *Salmonella* was isolated from 100% of larvae from group C-6.

In larvae groups P-3 and P-4, there was no increase in the phage titres over 72 h (Figure 3e) and phage numbers were comparable to the larvae groups only administered the respective phage cocktails (C-4 and C-5). In contrast, in the larvae group P-2 total phage abundance was ten-fold lower in comparison to the phage control group (C-3). However, after 48 h phage numbers increased ten-fold, suggesting active phage replication had occurred.

#### 2.6.2. Simultaneous Phage Treatment of Infected Larvae

Larvae co-infected with two (CoI-2), three (CoI-3) and four-phage (CoI-4) cocktails had over 88% survival rates after 24 h, which was similar to the infected control group C-6 (Figure 3b,d; Table 4). There were, however, significant reductions in *Salmonella* abundance in all larvae groups treated with phage cocktails, in comparison to C-6 (*p* ≤ 0.001).

After 48 h the survival rate of larvae in group C-6 dropped to 60%. In challenged, phage treated groups CoI-2, CoI-3 and CoI-4, the survival rates were 84%, 72% and 78%, respectively. This increased survival rate was reflected by significant reductions in *Salmonella* counts per larva in all phage treated groups compared to C-6 (*p* < 0.001). Average *Salmonella* counts were 7.4 × 10^2^, 2.5 × 10^2^, 7.5 × 10^1^ and 4.0 × 10^6^ CFU/larva for groups CoI-2, CoI-3, CoI-4 and C-6, respectively. *Salmonella* was not isolated from 50%, 60% and 55% larvae in groups CoI-2, CoI-3 and CoI-4, respectively.

After 72 h, all phage cocktails caused significant reductions in *Salmonella* (*p* < 0.001) in comparison to group C-6. The survival rate of the C-6 also dropped to 3%, compared to >65% in all phage treated groups (CoI-2, 3 and 4). In addition, *Salmonella* numbers were below the detection limit and were not isolated from 65%, 70% and 60% larvae in groups CoI-2, CoI-3 and CoI-4, respectively. Again, the data show phage treatment could clear infection in challenged larvae. Phage numbers remained constant for all phage treatment groups (Figure 3f) over 72 h, and there were no significant changes in phage numbers compared to phage control groups (C-3, C-4 and C-5).

#### 2.6.3. Sensitivity of Recovered *Salmonella* Colonies after Exposure to Phage Cocktails in a Larvae Model

**Figure 4:**
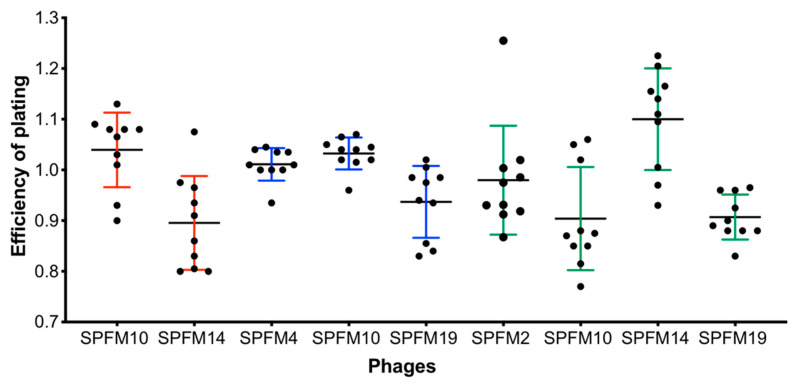
EOP of phages on *Salmonella* colonies isolated from the larvae infection model. Larvae were infected with the MDR S01160-12 *S*. Typhimurium strain and treated with either a 2-phage (SPFM10 and SPFM14), 3-phage (SPFM4, SPFM10 and SPFM19) or 4-phage cocktail (SPFM2, SPFM10, SPFM14 and SPFM19). If *Salmonella* colonies were recovered after 72 h, colonies were picked and for 10 colonies from each group phage EOP was assessed to the phages in the cocktails. Red error bars represent colonies picked after larvae were treated with the 2-phage cocktail, blue error bars for the 3-phage cocktail and green error bars for the 4-phage cocktail. Average plaquing efficiencies are shown from three biological replicates and error bars represent SEM.

To determine if phage resistance evolves in *G. mellonella*, *Salmonella* colonies recovered from infected larvae after 72 h were isolated and tested for susceptibility to phages they had been subjected to. All colonies recovered from both dead and living larvae remained sensitive to the phages (Supplementary data Tables S4–S6). For further analysis, ten *Salmonella* colonies from each phage treatment were randomly selected and the EOP of the individual phages within the cocktails was determined and compared to the wild-type *S.* Typhimurium S01160-12 strain (Figure 4). EOP values were ~1.0, indicating EOP was the same as on the wild-type strain and phage-resistant colonies were not isolated.

## 3. Discussion

In this study, we fully characterised six broad host range phages with the aim of developing a cocktail to prevent or treat *Salmonella* infection in pigs. All six phages have similar burst sizes and use a component on the LPS as their bacterial receptor. However, they are differentially sensitive to temperature and pH.

The temperature stability data showed the SPFM phages had distinct phenotypic differences. All phages were stable at 50 °C, consistent with other characterised phages [39,40]; however, SPFM10 was significantly more stable, remaining active even after being exposed to 90 °C for an hour. This is a very unusual phenotype, as, typically, when phages are exposed to high temperatures, they lose infectivity [41]. Clearly, an increased heat stability expands downstream phage applications as SPFM10 is likely to remain stable during spray drying to make dry phage powders, which would improve shelf life [42,43,44].

Phages SPFM10, SPFM14 and SPFM17 were less stable at pH 3 than SPFM2, SPFM4 and SPFM19. The pH stability of phages impacts the delivery method used in pigs, as if administered orally they will encounter acidic environments as they pass through the digestive tract of the pig [45]. The SPFM phages all remained infective above pH 4, but at lower pH, they may not survive the digestive tract in their native state. However, methods are being developed to encapsulate phages in polymers, to improve their stability at lower pH [46].

Ideally, phage or phage cocktails for therapeutic use will effectively eliminate multiple strains of the target bacterial species and delay occurrence of phage-resistant mutants [47]. To identify such phage(s), we tested the activity of six SPFM phages alone and in combinations in vitro. Phage cocktails were more effective than individual phages at bacterial lysis and lowered the re-growth of *S*. Typhimurium compared to when individual phages are used, presumably because there was less development of phage resistance. Thirty-six different phage combinations were tested in vitro and their ability to reduce bacterial cell numbers varied significantly. Although cocktails with three and four phages in were tested, it was cocktails with only two phages (SPFM10-SPFM14 and SPFM2-SPFM10) that were the most effective and reduced bacterial numbers by ~1 × 10^4^ CFU/mL within 2 h. In comparison, other studies have shown lower reductions in bacterial numbers, where a three-phage [48] and a six-phage cocktail [12] reduced *Salmonella* numbers by ~1 × 10^2^ CFU/mL. Our study highlights the importance of assessing different phage combinations to determine the optimal combinations to reduce the target pathogen.

After identifying the best phage cocktails in vitro, the efficacy of a two, three and four phage cocktails was tested in the in vivo larvae infection model. Our data showed that there was a correlation between in vitro and in vivo data as again the two-phage cocktail SPFM10-SPFM14 was the most efficient at reducing *Salmonella* numbers. The cocktail was more efficacious when it was administered prophylactically, where larvae survival rates after 72 h were 90% versus 72%, when the phage cocktail was administered concurrently with the larvae challenge. Furthermore, *Salmonella* was not isolated from over 65% of larvae, under either treatment regimen in larvae administered the two-phage cocktail, suggesting pathogen clearance. The fact that the prophylactic treatment was more effective than concurrent treatment is in keeping with other studies that found similar results in *C. difficile* [49] and *Pseudomonas* [34]. The increased phage efficacy with prophylactic treatment could be due to phages being distributed throughout the haemolymph before bacterial cells are introduced, which limits bacterial colonisation and contributes to larvae survival [34]. In contrast, in the co-infection model, the bacterial cells have more of an opportunity to colonise the haemolymph and thus the phages are less effective. *Salmonella* colonies isolated post-exposure were still sensitive to phage infection, as has been reported in other larvae challenge model studies [32,49,50,51]. However, it could also be possible the low challenge dose of 10^5^ CFU/larva used was insufficient to isolate phage resistant mutants. Moving forward resistant strains could have been potentially isolated if a higher dose of 10^7^ CFU/mL (as used in the killing assays) was used. Overall, our data on the efficacy of prophylactic phage treatment suggest it could be an important and testable intervention point to prevent bacterial infection in pigs.

A major message from this work is that there are considerable phenotypic differences (pH and temperature stability) between the phages despite them sharing 99% ANI. This observation is of fundamental importance to the way in which we understand phage biology and the strikingly few SNPs with distinct phenotypes will ultimately allow a better mechanistic understanding of these phages. Here, we discuss the types of the genetic changes and how they may be linked to phenotype.

The most striking phenotypic difference between the phages is heat stability. It is currently unclear why SPFM10 is more heat stable than the other phages tested. One possible explanation would be the environment from which they are isolated; however, it should be noted that SPFM4 and SPFM10 were isolated from the same source (food processing plant in Essex, UK), and yet their heat stability differs significantly. Therefore, it could be concluded that the evolution of the heat resistant phenotype is more specialised and complex than simply reflecting the environmental source.

Through SNP analysis, we identified that the heat stable phage SPFM10 has only one SNP difference to the heat labile phage SPFM14 (at 70,890 bp, locus tag SPFM10_00081), the same difference is seen between SPFM10 and SPFM2, 4, 17 and 19. Thus, we predict a single amino acid change could determine increased heat resistance. The function of this protein (locus tag SPFM10_00081) is unknown but the gene encoding it was previously identified to be under positive selection [35]. The protein was analysed for predicted secondary and tertiary structure using the programs JPred [52], Phmmer [53] and Phyre2 [54]. Phyre2 analysis identified a domain within the protein with similarity to an Fibronectin type III (FN3)/FN3-like domain (with 94–96% confidence). These domains are commonly found in eukaryotes and bacterial extracellular glycohydrolases [55,56]. FN3 domains are thought to contribute to bacterial recognition by facilitating binding to bacterial surface adhesion proteins [57]. Within phages, these domains are typically found within structural proteins of tailed dsDNA phages. For example, an analysis by Fraser et al. showed that FN3 domains were predominantly found in baseplate wedge initiator proteins, major tail proteins, and tail fibre proteins [58]. We hypothesise this protein is therefore a structural protein, likely to be linked with the tail or baseplate wedge. The conservative amino acid substitution (Thr to Ser) observed between the heat stable and heat labile versions of this protein is shown to elongate a predicted β-strand. Future structural studies of this protein and its variants could confirm whether the increased stability is directly linked to the protein and its secondary structure, as predicted from virtual models, or elucidate other causes for thermostability including altered interactions with other parts of the phage capsid/tail or even with bacterial surface adhesion proteins.

Both SPFM17 and SPFM10 have increased stability compared to other phages, yet only SPFM10 has a mutation of A -> T at position 70,890. Therefore, it is likely that one or more of the four other mutations in SPFM17 also provide increased resistance to heat compared to other phages, but not to the same degree as the mutation at position 70,890 bp. It should also be noted that the cost of heat resistance comes at the cost of increased sensitivity to acidic pH. Further work is needed to ultimately confirm which SNPs are linked to phenotypic differences and establish their mechanistic basis of action. Another study recently demonstrated that single nucleotide variations can increase the temperature stability of phages infecting *Pectobacterium* and *Ralstonia* [59], which further support our results that SNPs in key genes can have dramatic phenotypic effects.

The marked phenotypic differences of closely related phages were unexpected, with a single-base mutation the most likely cause of increased temperature stability and decreased pH stability. The likely single-base change that causes drastic phenotypic differences is an important consideration when using phages for practical purposes in pathogen control and treatment. There is limited information on the rates of mutation in bacteriophages with estimates for dsDNA phages of between 1.88 × 10^−4^ and 1 × 10^−7^ substitution rate per site per year [60]. For genomes of ~250 kb this equates to between ~0.24 and 45 substitutions per year [61]. How this rate of substitution holds true for this phage type during mass production is unknown, but it is clear that the heat stable phenotype should be assessed during production to ensure it is still present. Quality control procedures to monitor differences in batches would be required. As a scenario where a small proportion of the population have a new fixed mutation, leads to differences in efficiency between batches which could be negative or positive.

## 4. Materials and Methods

### 4.1. Bacterial Strains and Growth Conditions

Two *Salmonella enterica* subsp. Enterica serovar Typhimurium strains were used in this study: SL1344 (accession number FQ312003), a laboratory reference strain, and strain S01160-12 phage type DT193. The MDR *Salmonella* strain S01160-12 was isolated by the Animal Plant Health Agency, UK from an infected pig in 2012 [38] and is resistant to antibiotics: tetracycline, neomycin, ampicillin, sulfamethoxazole, chloramphenicol, gentamicin, streptomycin, compound sulphonamide and apramycin. Both *Salmonella* isolates were routinely grown on Xylose Lysine Deoxycholate (XLD) agar (Oxoid, Basingstoke, UK) for 18 h at 37 °C. To prepare liquid cultures, strains were inoculated in NZCYM broth (Melford Biolaboratories Ltd., Ipswich, UK) and grown for 18 h at 37 °C at 100 rpm.

### 4.2. Phages and their Genome Accession Numbers

Phages SPFM2, SPFM4, SPFM10, SPFM14, SPFM17 and SPFM19 from our *Salmonella* jumbo phage collection were used in this study [35]. Phages were previously deposited to European Nucleotide Archive and their GenBank accession numbers are: SPFM2 (LR535921.1), SPFM4 (LR535902.1), SPFM10 (LR535908.1), SPFM14 (LR535912.1), SPFM17 (LR535914.1) and SPFM19 (LR535916.1).

### 4.3. Phage Propagation and Titration

Phages were propagated via liquid propagation in NZCYM media as previously described in detail [35].

### 4.4. One-Step Growth Assay

A previously described method was followed [62]. Exponential cultures of SL1344 at optical density (OD_600_) 0.2, with cell densities of 10^7^ CFU/mL were mixed with phage at a multiplicity of infection (MOI) of 0.01. Phages were allowed to adsorb for 5 min at 37 °C and unbound phages were removed by centrifuging at 4200× *g* for 10 min. The bacterial pellet was re-suspended with NZCYM broth and incubated at 37 °C with shaking (100 rpm). Aliquots were taken every 10 min for 1.5 h and the small drop plaque assay method was used to determine the PFU/mL count [63]. Three biological replicates were performed each with three technical repeats.

### 4.5. Phage Receptor Analysis

Professor Sangryeol Ryu (Seoul National University, Seoul, South Korea) kindly sent us *S.* Typhimurium SL1344 strains with deletions in the flagellar production gene (SL1344 *flgK*); the gene that encodes vitamin B12 uptake outer membrane protein (SL1344 *butB*); and an RfaL ligase mutant the prevents LPS production (SL1344 *rfaL*) [37]. To determine if the phages use either of the three as their host receptor, phage lysates at titres ~5 × 10^9^ PFU/mL were diluted 10-fold and plated on the mutant strains using the small drop plaque assay method [63]. For the efficiency of plating (EOP) study three biological replicates were performed each with three technical repeats and EOP was compared to the wild-type strain SL1344.

### 4.6. Temperature and pH Stability Assays

Heat stability of phage lysate (volume 500 μL in NZCYM broth) at titre ~10^9^ PFU/mL was tested by exposing lysates to a range of temperatures of 4, 10, 20, 30, 40, 50, 60, 70, 80, 90 and 100 °C for one hour using a thermocycler (Bioline, UK). For pH stability, 100 μL of phage lysate was added to 900 μL of SM buffer at pH of 1, 2, 3, 4, 5, 6, 7, 8, 9, 10, 11, 12, 13 and 14. After this, the phage samples were incubated at room temperature for one hour. To determine phage titre after exposure to different temperatures and pH, the phage lysate was immediately serially diluted 10-fold and the small drop plaque assay method was used on LB 1% (*w*/*v*) agar plates with bacterial lawns of SL1344 [63]. Final phage titres were expressed as PFU/mL and three biological replicates were performed, each with three technical repeats.

### 4.7. Single-Nucleotide Polymorphism (SNP) and Indels Analysis

SNPs were detected by aligning reads against the reference genome SPFM10 with minimap2 using the short read settings. Resultant BAM files were processed with samtools v1.10 [64] to produce a mpileup file, used as input for VarScan v2.3 [65]. SNPs were called with ‘--min-var-freq 0.9, --min-avg-qual 20, --*p*-value’, with other settings left as default.

### 4.8. In Vitro Bacterial Killing Assay with Individual Phages and Phage Cocktails

Killing assays were conducted with individual phages and with different phage cocktail combinations (Table 4). The *S.* Typhimurium strain S01160-12 was chosen for these experiments as the strain is a representative MDR circulating in pig farms and all six phages are able to lyse the strain with comparable EOP to their host strain SL1344 [35]. For all killing assays, cultures of S01160-12 were grown to an optical density (OD_600_) of 0.2 (~10^7^ CFU/mL) at which point phage or phages were added at a MOI of 100 (phages were mixed at equal volumes at a final titres of 10^9^ PFU/mL). All killing assay experiments included a bacterial only control. Both the control and test samples were incubated at 37 °C with shaking (100 rpm), and aliquots were taken at the following time points: 0, 1, 2, 3, 4, 5 and 6 h. For each time point, bacterial concentrations (CFU/mL) were determined on LB 1% agar plates and phage concentrations (PFU/mL) by the small drop plaque assay method [63].

### 4.9. Testing Efficacy of Phage Cocktails in the In Vivo Galleria Mellonella Infection Model

#### 4.9.1. Preparation of Galleria Mellonella

Larvae were purchased from Live Food UK Ltd. (Rooks Bridge, UK), stored at 4 °C and used within two days. For all in vivo experiments larva that weighed approximately 0.25 to 0.30 g were chosen and were surface sterilised with cotton swabs dipped in 70% ethanol.

#### 4.9.2. *Salmonella* Infected *G. mellonella* Treated with Phage Cocktails

For all larvae phage therapy studies, previously described methods were used [32,38,49]. Both the *Salmonella* strain S01160-12 (LD_50_ dose of 10^5^ CFU) and phage cocktails at a final concentration of 10^7^ PFU (MOI 100 and phages were mixed at equal volumes) were administered to the larvae via the oral route using a Hamilton syringe pump. The strain S01160-12 was prepared by growing a liquid culture of S01160-12 overnight. The concentration of the cells in the culture was ~10^9^ CFU/mL and the culture was centrifuged at 4200× *g* for 10 min. The supernatant was discarded; the pellet was resuspended in 0.1 M phosphate-buffered-saline (PBS) and centrifuged at 4200× *g* for 10 min. The supernatant was discarded again; the pellet was resuspended in PBS and diluted 10-fold in PBS to 10^7^ CFU/mL. Larvae were administered 10 μL of bacterial culture, which equated to a final concentration of ~10^5^ CFU.

Phage cocktails were either administered prophylactically, 1 h prior to *Salmonella* infection or simultaneously with *Salmonella* (the regimens are detailed in Table 4. The latter method was conducted by first injecting larvae with *Salmonella* and then rapidly infecting larvae with the phage cocktail in a separate injection. For both regimens phage cocktails were administered at an MOI of 100 (final concentration of 10^7^ PFU). Larvae were incubated at 37 °C for 3 days. Every 24 h, survival of larvae was monitored, and larvae were sacrificed to determine CFU and PFU counts. For this, larvae were stored at −20 °C for 3 h, dissected to remove their haemolymph, which was suspended in 1 mL of PBS and vortexed for 30 s. To determine the PFU counts the samples were centrifuged at 5000× *g* for 5 min. The supernatant was then diluted 10-fold and titred by the small drop plaque assay method [63]. To determine the CFU counts the suspension was diluted with 10-fold dilutions and spot tested on XLD agar plates. For both the CFU and PFU counts, three technical repeats were conducted.

For each group listed in Table 2, 60 larvae were used per group and every 24 h 20 larvae were assessed for survival and dissected. If the larvae were unresponsive to touch and changed colour from light brown to black they were considered dead. Control larvae groups were included in all experiments and the groups were: healthy larvae; larvae only administered 10^7^ PFU phages via the oral route; larvae only administered with 10^5^ CFU S01160-12 via the oral route; and larvae administered PBS via the oral route (Table 2). In all control groups 60 larvae were used in total and 20 were scarified every 24 h to determine CFU and PFU counts in their haemolymphs.

#### 4.9.3. Larvae Survival Curves and Statistical Analysis

Three biological replicates were conducted for all larvae experiments, and survival data were plotted using Graphpad Prism version 6 (GraphPad Software Inc., San Diego, CA, USA) with the Kaplan–Meier method. The differences in survival rates were assessed using the Log-rank (Mantel-Cox) test.

#### 4.9.4. Testing Development of Phage Resistance In Vivo

*Salmonella* colonies recovered from the larva’s haemolymph in the phage treatment groups were screened to determine if resistance had developed to the respective cocktails they were exposed to. *Salmonella* colonies recovered from dead and alive larvae were picked and streaked on XLD agar plates. Plates were incubated at 37 °C for 18 h and then re-streaked onto XLD agar plates. The process was repeated three times. In total 60 colonies from each phage treatment were picked (Supplementary Tables S4–S6).

*Salmonella* colonies were inoculated into NZCYM broth and grown for 2 h at 37 °C at 100 rpm, prior to being used to make lawns to determine their sensitivity to phages via spot tests. Then, 10 µL of phages was spotted in triplicate at a concentration of 10^8^ PFU/mL. Further resistant studies were conducted by randomly selecting 10/60 colonies from each phage treatment and determining EOP of the phages on these strains compared to the wild-type strain S01160-12. In the EOP studies, phage lysates at a concentration of 10^8^ PFU/mL were diluted 10-fold and plated using the small drop plaque assay method [63]. For all EOP studies and spot tests, three biological and three technical replicates were conducted. EOP values were compared to EOP of the phages on the wild-type strain.

### 4.10. Statistical Analysis

Data presented and discussed in this study are average values from three biological replicates, of which each had three technical repeats. To determine the significance of bacterial and phages counts between treatments student *t*-tests were conducted. Significant differences (*p* values) between treatments were determined by comparing the results in the test group with the results obtained for the correspondent control group for each different time point or condition tested. Values of *p* < 0.05 were considered as statistically significant (* *p* < 0.05, ** *p* < 0.01 and *** *p* < 0.001).

## 5. Conclusions

In this study, we found that genetically similar *Salmonella* phages have very different phenotypes even though SNP analysis highlighted one to five SNP differences. Future genetic and biochemical work in our laboratory will determine how these SNPs relate to the phenotypic differences between the phages. We showed the surprising observation that the genetically similar phages are more effective as two-phage cocktails rather than as single phages or three or four phage cocktails. We also show how a natural set of phages can act as a starting point to pinpoint specific genes in order to unravel the function of key phage proteins that determine phenotype.

## 6. Patents

The SPFM *Salmonella* phages are part of a Leicester patent, pending. United Kingdom Patent Application 1815483.1 and international application number PCT/GB2019/052695.

## Figures and Tables

**Table 1:** EOP on wild-type SL1344 strain versus receptor mutant strains of SL1344.

	Efficiency of Plating ^1^		
Phages	SL1344 Δ*butB*	SL1344 Δ*flgK*	SL1344 Δ*rfaL*
SPFM2	1.00	0.99	0
SPFM4	0.99	1.00	0
SPFM10	0.98	1.00	0
SPFM14	1.00	1.00	0
SPFM17	0.99	1.00	0
SPFM19	1.00	1.00	0

^1^ Plaquing efficiencies of 1.00 show there is no difference in EOP on the wild-type strain versus the mutant strain.

**Table 2:** Phage SPFM10 was compared to SPFM2, SPFM4, SPFM14, SPFM17 and SPFM19 to identify SNPs between the phages.

Phage	Position in the Genome of SPFM10 (bp)	Locus Tag in SPFM10 ^1^	Protein Annotation	Base Change	Amino Acid Change	*p* Value
SPFM2	70890	SPFM10_00081	Hypothetical protein	A -> T	Thr -> Ser	1.05 × 10^−38^
	74294	SPFM10_00084	Hypothetical protein	A -> C	Ser -> Leu	1.70 × 10^−43^
	74298	SPFM10_00084	Hypothetical protein	G -> T	Ser -> Leu	4.28 × 10^−44^
SPFM4	70890	SPFM10_00081	Hypothetical protein	A -> T	Thr -> Ser	2.20 × 10^−144^
	74291	SPFM10_00084	Hypothetical protein	A -> G	Pro -> Leu	1.34 × 10^−238^
	74295	SPFM10_00084	Hypothetical protein	G -> T	Ser -> Arg	3.22 × 10^−247^
SPFM14	70890	SPFM10_00081	Hypothetical protein	A -> T	Thr -> Ser	5.14 × 10^−16^
SPFM17	57985	SPFM10_00067	Chromosome partition protein Smc	A- > G	Gin -> Arg	1.73 × 10^−49^
	70890	SPFM10_00081	Hypothetical protein	A -> T	Thr -> Ser	2.77 × 10^−66^
	74291	SPFM10_00084	Hypothetical protein	G -> A	Pro -> Leu	6.66 × 10^−94^
	74295	SPFM10_00084	Hypothetical protein	T -> G	Ser -> Arg	1.06 × 10^−92^
	113292	SPFM10_00140	Hypothetical protein	A -> G	Cys -> Cys	1.10 × 10^−65^
SPFM19	70890	SPFM10_00081	Hypothetical protein	A- > T	Thr -> Ser	1.55 × 10^−50^
	72949	SPFM10_00083	Hypothetical protein	C -> T	Ser -> Gly	1.74 × 10^−52^

^1^ Corresponding protein sequences from the genome of SPFM10 are listed in Supplementary Table S2.

**Table 3:** A list of phage and phage cocktails tested in in vitro killing assays.

	Phage or Phages in Cocktail
Single phages	SPFM2
	SPFM4
	SPFM10
	SPFM14
	SPFM17
	SPFM19
Two-phage cocktails	SPFM2-SPFM14
	SPFM4-SPFM10
	SPFM4-SPFM14
	SPFM4-SPFM17
	SPFM4-SPFM19
	SPFM10-SPFM14
	SPFM14-SPFM17
	SPFM14-SPFM19
	SPFM2-SPFM4
	SPFM2-SPFM10
	SPFM2-SPFM17
	SPFM2-SPFM19
	SPFM10-SPFM17
	SPFM10-SPFM19
	SPFM17-SPFM19
Three-phage cocktails	SPFM4-SPFM14-SPFM17
	SPFM4-SPFM14-SPFM19
	SPFM2-SPFM4-SPFM14
	SPFM2-SPFM14-SPFM17
	SPFM10-SPFM14-SPFM19
	SPFM4-SPFM17-SPFM19
	SPFM2-SPFM4-SPFM17
	SPFM4-SPFM10-SPFM19
	SPFM2-SPFM4-SPFM19
	SPFM10-SPFM17-SPFM19
	SPFM2-SPFM10-SPFM19
Four-phage cocktails	SPFM10-SPFM14-SPFM17-SPFM19
	SPFM2-SPFM10-SPFM14-SPFM19
	SPFM2-SPFM4-SPFM10-SPFM19
	SPFM2-SPFM10-SPFM17-SPFM19

**Table 4:** The different larvae groups used to evaluate the effectiveness of phage therapy for the treatment of challenged larvae.

Groups ^1^	Description of Larvae Groups ^2^
Controls	
C-1	Healthy larvae
C-2	Administered PBS
C-3	Administered the 2-phage cocktail SPFM10-SPFM14
C-4	Administered the 3-phage cocktail SPFM4-SPFM10-SPFM19
C-5	Administered the 4-phage cocktail SPFM2-SPFM10-SPFM14-SPFM19
C-6	Challenged with *Salmonella*
Prophylactic phage treatments	
P-2	Administered 2-phage cocktail SPFM10-SPFM14 one hour prior to challenge with *Salmonella*
P-3	Administered 3-phage cocktail SPFM4-SPFM10-SPFM19 one hour prior to challenge with *Salmonella*
P-4	Administered 4-phage cocktail SPFM2-SPFM10-SPFM14-SPFM19 one hour prior to challenge with *Salmonella*
Co-infection studies	
CoI-2	Administered 2-phage cocktail SPFM10-SPFM14 simultaneously with *Salmonella*
CoI-3	Administered 3-phage cocktail SPFM4-SPFM10-SPFM19 simultaneously with *Salmonella*
CoI-4	Administered 4-phage cocktail SPFM2-SPFM10-SPFM14-SPFM19 simultaneously with *Salmonella*

^1^ In each group 60 larvae were used for the study. Every 24 h 20 larvae were checked for survival and culled to enumerate PFU and CFU counts. ^2^ Phages, challenge with MDR *S.* Typhimurium S01160-12 strain and PBS were administered orally.

## Data Availability

The data presented in this study are available in article and supplementary material.

## Supplementary Materials

The following supporting information can be downloaded at: https://www.mdpi.com/article/10.3390/ph15010058/s1, Supplementary Table S1: Latent period and burst sizes of SPFM phages. Supplementary Table S2: Protein sequences of the genes in which SNPs were identified in the genome of SPFM10 when compared to phage genomes SPFM2, 4, 14, 17 and 19. Supplementary Table S3: Average *Salmonella* counts following infection with phage or phage cocktail in killing assay experiments. Supplementary Table S4: sensitivity of colonies picked after being exposed to the two-phage cocktail SPFM10-SPFM14 in the challenge larvae experiments, to individual phages within the cocktail. Supplementary Table S5: sensitivity of colonies picked after being exposed to the three-phage cocktail SPFM4-SPFM10-SPFM19 in the challenge larvae experiments, to individual phages within the cocktail. Supplementary Table S6: sensitivity of colonies picked after being exposed to the four-phage cocktail SPFM2-SPFM10-SPFM14-SPFM19 in the challenge larvae experiments, to individual phages within the cocktail.
